# Bilateral Digital Extensor Hypoplasia Correction: A Case Report and Systematic Review

**DOI:** 10.1055/s-0042-1757311

**Published:** 2023-02-01

**Authors:** Marudeen Aivaz, Esperanza Mantilla-Rivas, Ashleigh Brennan, John Thomas, Elizabeth L. Malphrus, Monica Manrique, Albert K. Oh, Gary F. Rogers

**Affiliations:** 1 Division of Plastic and Reconstructive Surgery, Children's National Hospital, Washington, District of Columbia

**Keywords:** extensors tendons, congenital hypoplasia, extensor retinaculum, extensor tendon transfer

## Abstract

Digital extensor hypoplasia (DEH) is a rare malformation that presents with loss of active finger extension at the metacarpophalangeal (MCP) joints. Descriptions of optimal treatment and outcomes in this population are sparse. We describe successful operative treatment of a child with DEH involving the extensor digitorum communis, extensor digiti minimi, and the extensor indicis proprius tendons. The 5-year-old male patient was referred for severe limitation on bilateral finger extension since birth. He had been previously diagnosed with arthrogryposis and managed conservatively. Due to lack of improvement, magnetic resonance imaging was done evidencing hypoplasia/aplasia of the extensor tendons. The patient underwent successful tendon transfers using extensor carpi radialis longus to the common extensor tendons, and one hand required an additional tenolysis procedure. 2 years postoperatively, his MCP position and finger extension are markedly improved, and he is able to grip objects without limitation or difficulty. The patient returned to full activity without restriction.

## Introduction

Variations in the anatomy of the hand extensor tendons are common and may include duplication, aplasia, or anomalous insertions and origins. 1 Hypoplasia of the finger extensor tendons is a rare congenital malformation first described in 1934 by Zadek but poorly characterized in the literature. 2 Findings include underdevelopment of some or all of the finger extensors tendons, adherence of the extensor tendons to the underlying periosteum as in our case, and atrophy or underdevelopment of the associated muscle. 3 We report the clinical findings and successful operative treatment of a child with isolated bilateral hypoplasia involving the extensor digitorum communis (EDC), extensor digiti minimi (EDM), and the extensor indicis proprius (EIP) tendons.

## Case

**Fig. 1:**
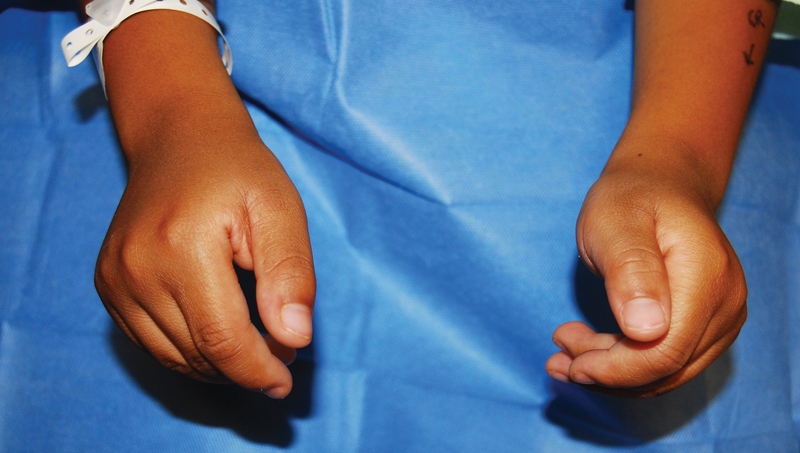
A case of bilateral digital extensor hypoplasia. Preoperative picture demonstrating no active extension of the index, middle, ring, and small finger at the metacarpophalangeal (MCP) joint.

**Fig. 2:**
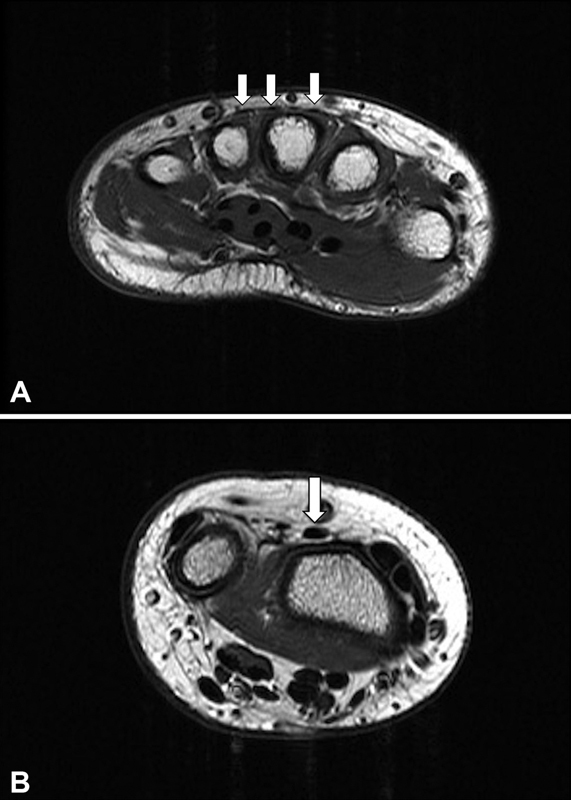
Left axial T1 magnetic resonance image. ( **A** ) Level of metacarpals showing hypoplasia of the extensor digitorum tendons. ( **B** ) Level of distal radioulnar joint showing hypoplasia of the extensor digitorum communis (EDC), presence of normal size pollicis extensors tendons.

A 5-year-old boy was referred to our clinic for severe limitation of finger extension on both hands since birth. He had no significant past medical or family history. An orthopaedic surgeon diagnosed him with arthrogryposis at age 2 and recommended stretching and observation, but no improvement was noted. The patient's physical findings included resting flexed position (> 60 degrees) of the long, ring, and small fingers at the metacarpophalangeal (MCP) joints of both hands. The patient was unable to actively extend the MCP joints beyond –60 degrees regardless of wrist position but had full passive motion ( Fig. 1 ), and this severely limited his ability to grip cups and other larger objects. He was able to extend the proximal and distal interphalangeal joints normally, make a tight fist, and grip firmly. Wrist motion and strength were normal with no sensory deficits. Because the patient had no passive joint stiffness and no other affected joints, the diagnosis of arthrogryposis was questioned and a magnetic resonance imaging (MRI) scan without contrast of the left upper extremity demonstrated undeveloped and poorly defined EDC tendons and absence of the EDM tendon ( Fig. 2A and B ).

**Fig. 3:**
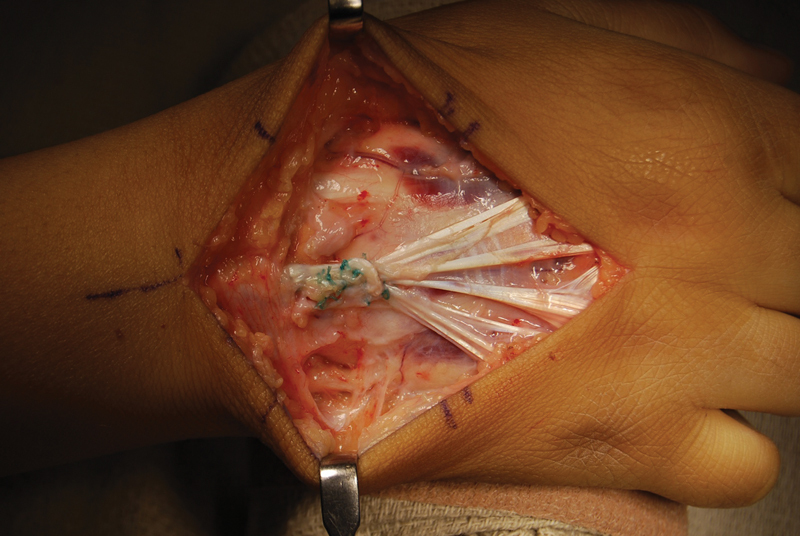
Intraoperative tendon transfer. Intraoperative picture demonstrating the extensor carpi radialis longus (ECRL) sutured to the extensor digitorum communis using a Pulvertaft weave.

Surgery was done at the age of 6 years; the extensor tendons of the left hand were exposed through a longitudinal dorsal hand incision extending from the long finger MCP joint to the distal forearm. There was clear hypoplasia of the common extensor, EIP, and EDM tendons, and all tendons were adherent to the underlying periosteum. The adhesions required meticulous elevation using sharp and blunt dissection, and tenolysis was required in all digits from the proximal interphalangeal (PIP) joint to the myotendinous junction. Two-thirds of the stenotic retinaculum was cut distally to allow better exposure and the tendons dissipated proximally into a fibro fatty vestige of each involved muscle. The extensor carpi radialis longus muscle (ECRL) was identified and, as suggested by MRI, was healthy and contracted readily with stimulation. The ECRL tendon was detached from its bony connection at the dorsal base of the 2nd metacarpal, rerouted into the 4th compartment, and sutured to the common extensor tendons using a Pulvertaft weave ( Fig. 3 ). The repair was tensioned to bring the finger MCP joints into full extension with the wrist in neutral extension. After repair, passive wrist motion confirmed finger-thumb opposition when the wrist was in maximal extension.

**Fig. 4:**
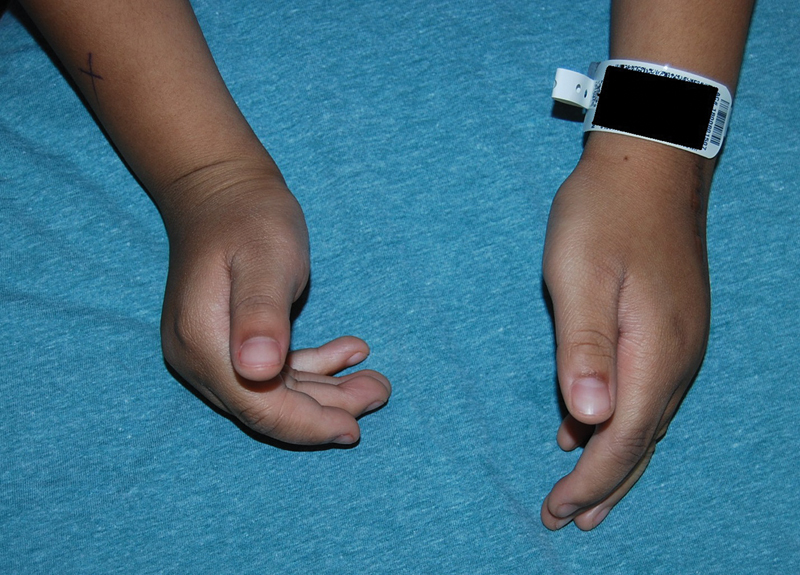
Postoperative results after tendon transfers. Twelve months postoperative result of the left hand with residual 15-degree extensor lag at (metacarpophalangeal [MCP]). Right hand with limited extension of the index, middle, ring, and small fingers at the MCP joint, before surgery.

The patient was placed in a short arm cast for 4 weeks after which he was allowed unrestricted activity and movement. One year after this procedure, he underwent tenolysis to improve active tendon excursion with good improvement ( Fig. 4 ). Five months later, the contralateral side was managed similarly, except the patient was allowed full, unrestricted activities and range of motion. Three months after the surgery, the patient has residual 15-degree extensor lag at the MCP joints but markedly improved function and ability to grasp small and large objects.

## Discussion

The etiopathogenesis of digital extensor hypoplasia (DEH) is unknown, but one widely accepted theory is that the tendon hypoplasia occurs due to decreased innervation of the extensor muscles in the forearm, similar to the etiology of certain forms of arthrogryposis. 4 5 6 While this proposal has some empirical support, Vartanian et al presented an affected patient with a normal electromyography and nerve conductions studies, indicating isolated muscle aplasia. 7 Treatment is focused on improving finger MCP extension and mobility ( Table 1 ). 8 9 10 11 12 13 14 15 16 17 Hand bracing has been reported to improve finger function and, similar to management of arthrogryposis, this treatment is most effective in younger patients. Most authors recommend starting with early bracing and therapy and progressing to tendon transfers if these modalities are unsuccessful. 12 These treatments may improve finger flexibility and static position, but it is inconceivable that they can restore active finger extension in a patient like ours who lacks a viable EDC muscle to power these movements. Our patient began splinting and hand therapy prior to the age of 2 with little benefit. For patients who do not respond to conservative treatment, tendon transfers can restore finger function.

**Table 1:** Review of surgical treatment in patients with extensor tendon hypoplasia

Case	Number of patients	Age (s)	Diagnoses	Diagnosis modality	Treatment	Reference
1	8	N/A	U/L and B/L absence or hypoplasia of EPB thumb extensors	Physical examination, intraoperative findings	N/A	White and Jensen (1952) 8
2	6	N/A	U/L and B/L hypoplasia of extensor tendons of digits and thumb	Physical examination	N/A	Crawford et al (1966) 4
3	5	6 wk, 6 wk, 11 wk, 2 mo, 4 mo, 4 mo, 7 mo, 2 y	U/L and B/L absence or hypoplasia of EPB thumb extensors	Physical examination	Plaster cast splinting, manipulation and stretching, extensor to extensor tendon graft	Weckesser et al (1968) 9
4	5	2, 3, 6, 9, 54	B/L or U/L hypoplasia of EDC, EIP, EPL, ECRL, ECB tendons or combination thereof	Intraoperative findings	Extensor to extensor graft, flexor to extensor graft	Tsuge (1975) 10
5	1	16	U/L and B/L hypoplasia of EDC and extensor pollicis longus	Intraoperative findings	Extensor to extensor graft	McMurtry and Jochims (1977) 6
6	3	2, 3, 5	U/L absence of EIP, EPL, EPB	Physical examination	Manipulation and stretching, extensor to extensor graft	Jochims (1983) 11
7	3	17, 19, 22	U/L hypoplasia or absence of EDC, EPB, EPL	Intraoperative findings, electromyography	Flexor to extensor graft	Hamanishi et al (1986) 5
8	1	18	U/L hypoplasia of EPL, EDC, and EDQ tendons	MR, intraoperative findings	Extensor to extensor graft	Vartany et al (1996) 3
9	1	8	U/L hypoplasia of EIP, EPL, EDC, and EDQ tendons	Intraoperative findings	Brachioradialis to extensor graft, extensor to extensor graft	Wajid and Rangan (2001) 12
10	1	7	U/L hypoplasia of EDC tendon and absence of EIP tendon (Escobar syndrome)	Intraoperative findings	Extensor to extensor graft	Aslani et al (2002) 13
11	1	12	B/L hypoplasia of EDC tendons	Intraoperative findings	Flexor to extensor graft	Tungshusakul et al (2011) 14
12	1	5	U/L absence of EIP and hypoplasia of EPL and EPB	Ultrasonography, intraoperative findings	Extensor to extensor graft	Gong et al (2012) 15
13	1	42	B/L absence of EIP tendons	Physical examination, ultrasonography, intraoperative findings	Flexor to extensor graft	Taylor and Casaletto (2017) 16
14	1	34	U/L hypoplasia of EDC tendon and extensor retinaculum	MR, intraoperative findings	Flexor to extensor graft	Vartanian et al (2020) 7
15	1	4	U/L absence of EPB tendon	Physical examination, intraoperative findings	Extensor to extensor graft	Serbest et al (2015) 17

Abbreviations: B/L, bilateral; ECRL, extensor carpi radialis longus; EDC, extensor digitorum communis; EDQ, extensor digiti quinti; EIP, extensor indicis proprius; EPB, extensor pollicis brevis; EPL, extensor pollicis longus; MR, magnetic resonance; U/L, unilateral.

While other authors have advocated using a flexor tendon to restore extensor function, 6 12 we chose the ECRL tendon as our donor muscle for the transfer for several reasons: it lies in the same operative field and requires no additional exposure to harvest, it is expendable, it is synergistic with the EDC function, and the muscle unit has excellent excursion and strength. Moreover, the direct line of pull makes proper tensioning of the reconstruction much easier than a more indirect flexor transfer that must be routed around the radial aspect of the distal forearm or passed through the interosseous membrane, which in a child can lead to adhesions or even ectopic bone formation. Because the muscle atrophy/agenesis inherent in this condition may not be isolated to the EDC muscle, it is imperative to ensure that the ECRL muscle is normal in appearance and contractility. If it is not, one must be prepared to move to a different donor. Other authors have used extensor carpi radialis brevis, 10 flexor digitorum superficialis, 4 7 and brachioradialis grafts. 12 In addition to tendon transfer, tenolysis of tendon adhesions and reconstruction of the extensor retinaculum may also be necessary during the initial procedure. 7 Our decision to immobilize the patient's fingers and wrist after the first transfer was due to his age and worries of compliance. This resulted in the need for another tenolysis procedure and, because of this, we elected for unrestricted mobilization after the transfer on the contralateral side with good results. The decision should hinge largely on the strength of the tendon repair, which was excellent in each instance.

Several other conditions can cause impaired finger extension at an early age. For example, congenital clasped thumb finger is characterized by persistent bilateral flexion and adducted posture of the thumb at the MCP joint. Similar to DEH, this entity is often caused by an abnormal extensor mechanism often accompanied with narrowing and contracture of the first web space. 18 Clasped thumb can be associated with different conditions including arthrogryposis, digitotalar dysmorphism, and Freeman-Sheldon syndrome, and it is usually classified into three types. Clasped thumb type I where the patient can passively abduct and extend against resistance of thumb flexors. Type II when there are associated hand contractures, and type III or rigid clasped thumb where there is marked soft-tissue deficits. 19 Treatment is usually conservative with serial splinting and stretching; however, surgery may be indicated in cases with contracture, persistent deformity, and significant soft tissue defects. 18 Camptodactyly is another condition that can resemble DEH. It is characterized by flexion contracture of the PIP joint in one or multiple digits. Treatment consists of observation with passive stretching in the majority of cases; nevertheless, surgical management is indicated in cases of progressive deformity leading to functional impairment. 20
